# Nasal Cannula Apneic Oxygenation Prevents Desaturation During Endotracheal Intubation: An Integrative Literature Review

**DOI:** 10.5811/westjem.2017.12.34699

**Published:** 2018-02-22

**Authors:** Joshua M. Gleason, Bill R. Christian, Erik D. Barton

**Affiliations:** * Ross University School of Medicine, Miramar, Florida; † University of California Irvine Health, Department of Emergency Medicine, Orange, California

## Abstract

Patients requiring emergency airway management may be at greater risk of acute hypoxemic events because of underlying lung pathology, high metabolic demands, insufficient respiratory drive, obesity, or the inability to protect their airway against aspiration. Emergency tracheal intubation is often required before complete information needed to assess the risk of procedural hypoxia is acquired (i.e., arterial blood gas level, hemoglobin value, or chest radiograph). During pre-oxygenation, administering high-flow nasal oxygen in addition to a non-rebreather face mask can significantly boost the effective inspired oxygen. Similarly, with the apnea created by rapid sequence intubation (RSI) procedures, the same high-flow nasal cannula can help maintain or increase oxygen saturation during efforts to secure the tube (oral intubation). Thus, the use of nasal oxygen during pre-oxygenation and continued during apnea can prevent hypoxia before and during intubation, extending safe apnea time, and improve first-pass success attempts. We conducted a literature review of nasal-cannula apneic oxygenation during intubation, focusing on two components: oxygen saturation during intubation, and oxygen desaturation time. We performed an electronic literature search from 1980 to November 2017, using PubMed, Elsevier, ScienceDirect, and EBSCO. We identified 14 studies that pointed toward the benefits of using nasal cannula during emergency intubation.

## INTRODUCTION

Oxygen desaturation below 70% puts patients at risk for dysrhythmia, hemoglobin decompensation, hypoxic brain injury, and death.^1^–^3^ The challenge for emergency physicians (EP) is to secure an endotracheal tube rapidly without critical hypoxia or aspiration.^1^ Preoxygenation prior to intubation extends the duration of “safe apnea” (the time it takes until a patient reaches an oxygen saturation level of 88% to 90%), to allow for placement of a definitive airway.^1^ Below that level, oxygen offloading from hemoglobin enters the steeper portion of the oxyhemoglobin dissociation curve, and can decrease to critical levels of oxygen saturation (<70%) within seconds.^1^

Alveoli will continue to take up oxygen even without diaphragmatic movements or lung expansion. Within some of the larger airways, turbulent flow could generate a cascade of turbulent vortex flows extending into smaller airways. Each vortex could communicate with the vortex above and below it, like a series of interlocking gears.^4^

The main goal of preoxygenation is to extend safe apnea time, which is more likely if certain physiological criteria are met (i.e., denitrogenation of the lungs and achieving an arterial oxyhemoglobin saturation (SaO_2_) of 100% as close as possible). Denitrogenation involves using oxygen to wash out the nitrogen contained in lungs after breathing room air, resulting in a larger alveolar oxygen reservoir. When breathing room air (79% nitrogen), 450 mL of oxygen is present in the lungs of an average healthy adult. When a patient breathes 100% oxygen, this washes out the nitrogen, increasing the oxygen in the lungs to 3,000 mL.

EPs and emergency medical services (EMS) use several devices to deliver oxygen or increased airflow to patients in respiratory need. Nasal cannula is used primarily for apneic oxygenation rather than pre-oxygenation. Previous recommendations were to place high-flow nasal cannula (HFNC) with an initial oxygen flow rate of 4 L/min, then increase to 15 L/min to provide apneic oxygenation once the patient is sedated. A nasal cannula can be placed above the face mask until just prior to attempting laryngoscopy, at which point it is placed in the nares to facilitate apneic oxygenation. The standard non-rebreather mask (NRB) delivers only 60% to 70% inspired oxygen (FiO_2_)at oxygen flow rates of 15 L/min. The FiO_2_ can be improved by connecting the NRB to 30–60 L/min oxygen flows from rates of 15 L/min. The use of NRBs is limited in patients with high inspiratory flow rates as FiO_2_ may be decreased due to NRB design (i.e.*,* seal, valve function). Some devices with effective seals and valves will collapse onto the patients face at high inspiratory flow rates causing transient airway obstruction.

A bag-valve mask (BVM) may approximate an anesthesia circuit for preoxygenation. BVMs vary in performance according to the type of BVM device, spontaneous ventilation vs. positive pressure ventilation, and the presence of a positive end-expository pressure (PEEP) valve. During spontaneous ventilation the patient must produce sufficient negative inspiratory pressures to activate the inspiratory valve. The negative pressures generated within the mask may lead to entrapment of room air and lower FiO_2_ during pre-oxygenation. A BVM’s performance increases during spontaneous breathing by administering high-flow oxygen, using a PEEP valve, and assisting spontaneous ventilations with positive pressure ventilations in synchrony with the patient’s spontaneous inspiratory efforts. Continuous positive airway pressure improves oxygenation by increasing functional residual capacity by reversing pulmonary shunting through the recruitment of poorly ventilated lung units.

**Figure 1:**
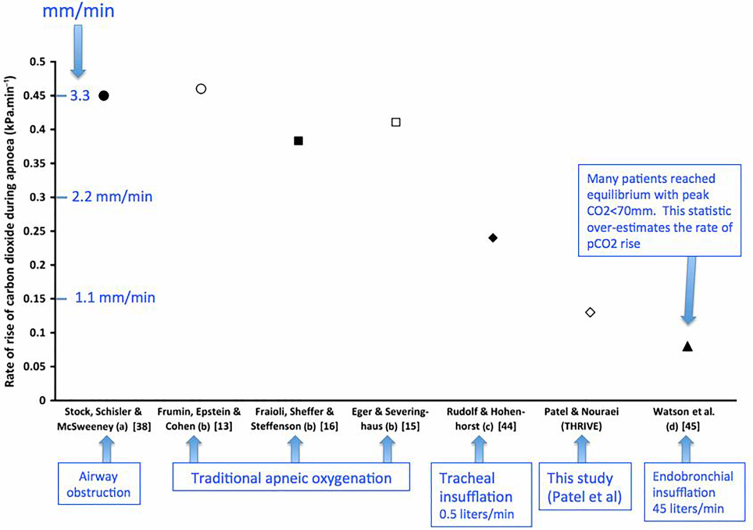
Rate of rise of carbon dioxide (CO_2_) levels during intubation under different apnea conditions undertaken within the study referred to (a) airway obstruction; (b) classical apneic oxygenation; (c) low-flow intra-tracheal cannula; and (d) high-flow intratracheal cannula.

In an apneic patient approximately 250 mL/minute of oxygen moves from the alveoli into the bloodstream. Conversely, only 8–20 mL/minute of carbon dioxide (CO_2_) moves into the alveoli during apnea with the remainder buffered in the bloodstream; this causes the net pressure in the alveoli to become slightly subatmospheric, generating a mass flow of gas from pharynx to alveoli via diffusion. 3 Regarding CO_2_ concentrations, Patel et al., 2015 provided evidence of those concentrations during apnea. Figure 1 shows the rate of CO_2_ concentration levels rising in various forms of apnea.^5^ It’s interesting to note that traditional apneic oxygenation has a similar rate of CO_2_ concentration rise when compared to airway obstruction.

High-flow oxygen therapy through a nasal cannula is a technique whereby oxygen is delivered to the nose at high flow rates. Higher flow rates generate low levels of positive pressure in the upper airways, and the fraction of FiO_2_ can be adjusted by changing the fraction of oxygen in the driving gas.^21^ The high flow rates may also decrease physiological dead space by flushing expired CO_2_ from the upper airway.^21^

**Population Health Research Capsule**

What do we already know about this issue?

During apnea created by rapid sequence intubation, high-flow nasal cannula maintains or increases oxygen saturation during efforts to secure an endotracheal tube.

What was the research question?

Does the use of high-flow nasal cannula during intubation prevent oxygenation desaturation?

What was the major finding of the study?

With additional studies, this study confirms that the use of nasal cannula during intubation prevents oxygen desaturation except in those with respiratory failure.

How does this improve population health?

Employing nasal oxygen during intubation can prevent hypoxia, extending safe apnea time, and improve first-pass success attempts.

The time period between becoming apneic and oxygenated via intubation is a vulnerable moment in the patient’s oxygen status, and can possibly be alleviated by using a nasal cannula. It was noted that traditional apneic oxygenation has a similar rise in CO_2_ concentration as an airway obstruction; so, can the use of nasal cannula during endotracheal intubation prevent oxygen desaturation? While various devices are used to preoxygenate patients, no standardized protocol exists. Despite the use of this technique by both anesthesiologists and EPs, to date implementing a nasal cannula during intubation has not been part of the standard of care in these procedures. The objective of this review was to evaluate studies that provide evidence for HFNC efficacy in preventing oxygen desaturation during intubation.

## METHODS

We identified articles from the following databases: PubMed, the National Center for Biotechnology Information, Elsevier, ScienceDirect, and EBSCO. The search was limited to articles in English published from 1980 – November 2017. We searched the following keywords: *nasal cannula, intubation, oxygen, hypoxia, hypoxemia, tracheal, pharyngeal, apnea, apneic, pre-oxygenation, insufflation*. We reviewed all abstracts to identify articles that assessed the usage of a nasal cannula during pre-oxygenation, apnea, and intubation. To ensure complete detection of all relevant studies, we cross-referenced all articles from the bibliography of the selected articles. After reviewing each article, we selected studies that met the following inclusion criteria: the use of nasal cannula or nasopharyngeal insufflation during intubation. We excluded studies that oxygenated patients with a NRB and/or BVM during periods of apnea. Past medical history and comorbidities were not taken into consideration. We evaluated studies by comparing the use of a nasal cannula or nasopharyngeal oxygen insufflation during intubation vs. the non-use of nasal cannula or nasopharyngeal oxygen insufflation; oxygen saturation levels before and during intubation, and in some groups time to desaturation. We determined oxygen saturation using two measurements: arterial SaO_2_ as determined by an arterial blood gas (ABG) test; and pulse oximetry as measured by peripheral oxygen saturation (SpO_2_).

## RESULTS

**Figure 2:**
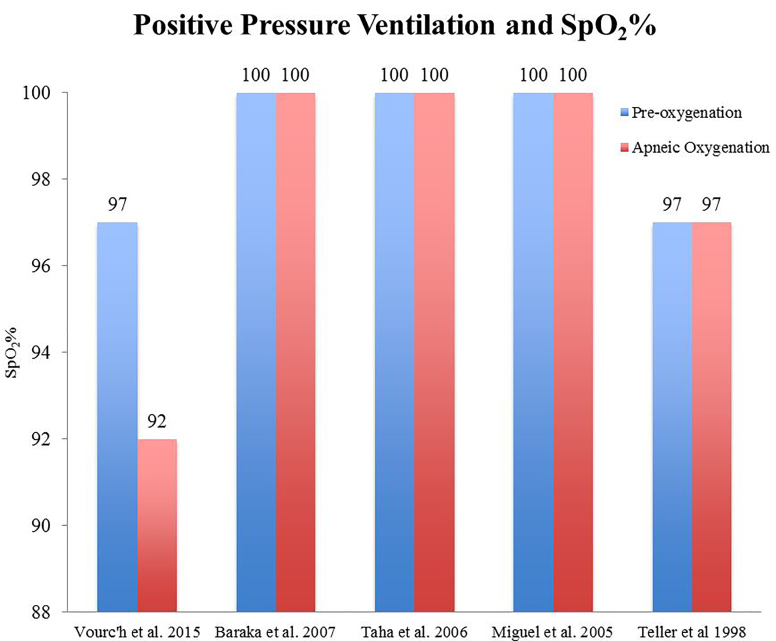
Positive pressure ventilation and peripheral oxygen saturation (SpO_2_) %. Patients were recorded on their initial SpO_2_% and lowest SpO_2_% during intubation. Each patient’s oxygen saturation level was raised before intubation to the respective blue lines before undergoing intubation with nasal cannula use. Red lines represent lowest SpO_2_ levels reached during intubation with nasal cannula usage. Vourc’h et al., 2015 reported a mean pre-oxygenation and median apneic oxygenation SpO_2_%, respectively.

To assess whether the use of nasal cannula during intubation would prevent oxygen desaturation, we compiled a list of studies (Figure 2) that report the mean or median oxygen saturation percentages (SpO_2_%) with this intervention. A baseline SpO_2_% was taken after each patient was appropriately raised above hypoxic levels (usually > 95%) during the standard preoxygenation, four-minute procedure of a BVM. SpO_2_% was then monitored during intubation. The intervention was compared against non-use to demonstrate its efficacy.

**Figure 3:**
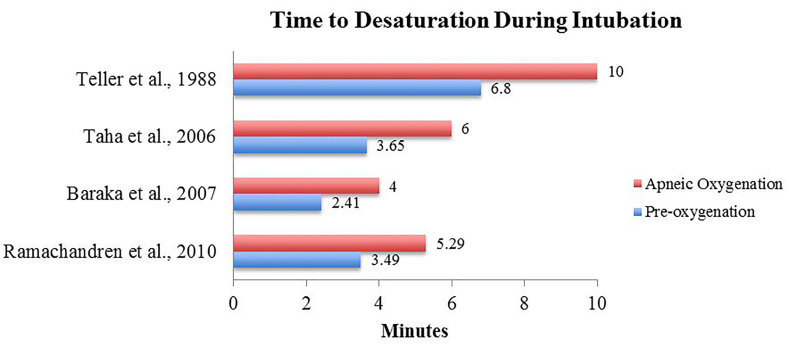
Time to desaturation during intubation. The control (without nasal cannula or blue line) and intervention group (w/ nasal cannula or red line) both underwent preoxygenation to peripheral oxygen saturation (SpO_2_) ranges of 92–100% and was timed in minutes when SpO_2_ level fell below various thresholds (range = 92–95%). Teller et al., 1988, Taha et al., 2006, and Baraka et al., 2007 had a maximum apneic cut-off limit of 10, 6, and 4 minutes.

Prior to intubation, several studies first examined the duration of desaturation occurrence by using nasal cannula. Figure 3 displays the time to desaturation before intubation. The intervention was compared against non-use to display an extension of safe apnea time during intubation. We identified 14 studies that investigated the efficacy of apneic oxygenation during intubation, including the use of nasal cannula. Evidence was compiled in PICO (Populations/people/patient/problem Interventions Comparison Outcome) format and displayed in Table. Of the 18 studies, four concluded that nasal cannula use did not prevent desaturation during intubation. 8, 9, 18, 23

## DISCUSSION

**Figure 4:**
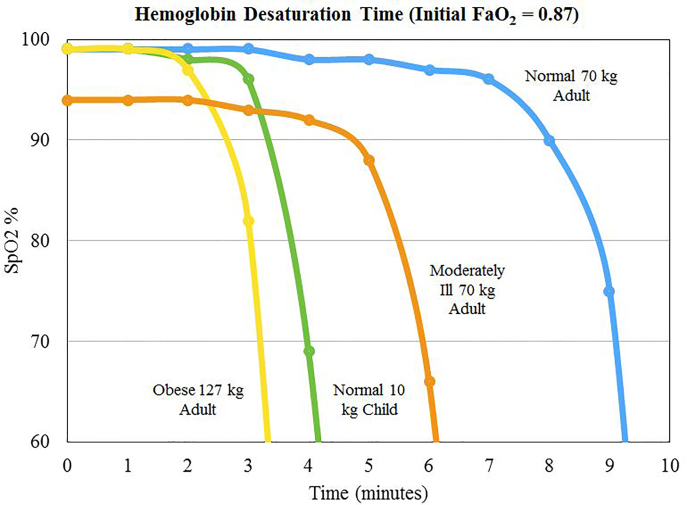
Hemoglobin desaturation time (initial FaO_2_ = 0.87). Adapted from Patel and Nouraei (2015). %SpO_2_ vs. time of apnea for various types of patients. FaO_2_, alveolar oxygenation fraction; SpO_2_, oxygen saturation

Desaturation in apneic patients undergoing rapid sequence intubation (RSI) procedures is predictable and reproduceable. In fact, desaturation rates are also determined by the patient’s underlying condition and body habitus (Figure 4). In periods of apnea or a completely obstructed airway, the time to desaturation is much shorter in obese adults and in children, demonstrated by a precipitous drop of hemoglobin oxygen levels after only 2.5–3.5 minutes. Depending on the emergency such as cardiac arrest or a trauma, which can affect cardiac output, the time to desaturation in such populations will likely be even shorter. The effects of hypoxemia can take place rather quickly: thus the need for quick intervention. The use of nasal cannula can effectively delay critical hemoglobin desaturation.^17^

In this paper, we examined 18 studies through a standardized literature search. Methodologically, all studies were performed with the same protocol of preoxygenation prior to and followed by nasal cannula use during intubation, lending more credence to its favorable results. Of those 18, 14 studies pointed towards the use of nasal cannula during intubation carrying benefits to the patient undergoing intubation, while nine studies reported an increase or maintenance of oxygen saturation levels. Despite patients having various medical conditions, nasal cannula use during intubation extended the duration of safe apnea. The gaps in current research include the following: the use of varying levels of oxygen flow such as 5L vs. 15L O_2_ and whether or not it is efficacious in diverse presenting medical conditions such as trauma, anaphylaxis, or other comorbidities.

Apneic oxygenation provides significant benefit in terms of improving SpO_2_ for most intubations. Miguel-Montanes et al., 2005 concluded that HFNC was found to be more effective than a NRB mask for preoxygenation in intensive care unit (ICU) patients by improving SpO_2_. It remains unclear how the use of HFNC compares with preoxygenation using a combination of standard nasal cannula and a NRB mask, or to the combination of standard nasal cannula and use of a BVM with a PEEP valve for apneic oxygenation. Although HFNC cannot compensate for ineffective preoxygenation, it may serve as a useful apneic oxygenation adjunct by extending safe apnea time. Further research is required to solidify or refute this consistent evidence.

While most studies concluded there was a benefit of apneic oxygenation to prevent desaturation during intubation, four studies found no benefit.^8^, ^9^, ^18^, ^23^ Of these, three are high-quality, randomized control trials and do not show statistical support.^8^, ^9^, ^18^ One should note the characteristics of the patients. The study population in Semler et al., 2015 were ICU patients requiring intubation, while those in Vourc’h et al., 2015 were in respiratory failure; this points toward no benefit when hypoxic respiratory failure is the indication for intubation.^26^ Similarly, in Caputo et al., 2017 the majority of patients in both the apneic oxygenation group (61 of 100) and standard-of-care group (59 of 100) were intubated due to a “pulmonary” indication, totaling 60% of the patient population. Considering the majority of intubations were performed due to “pulmonary” related causes, one would expect a non-significant result, which is consistent with Semler et al., 2015 and Vourc’h et al., 2015.

In contrast, the studies of patients undergoing elective surgery showed significant increases in time to oxygen desaturation, demonstrating that apneic oxygenation prior to intubation is only helpful in certain conditions, namely non-respiratory.^11^–^15^ While Caputo et al., 2017 analyzed apneic oxygenation from a broad mix of medical conditions and did not show statistical significance, it did not distinguish between the intervention’s efficacy in respiratory vs. non-respiratory causes as the results reflect all conditions (i.e., pulmonary, trauma, neurologic, cardiac, etc.). The reason that patients in respiratory failure or who are hypoxic prior to intubation do not benefit from apneic oxygenation is unclear. One hypothesis posits the development of pulmonary circulatory shunting, rendering passive ventilation ineffective.^26^

In light of Caputo et al., 2017, it continues to be confirmed that patients with respiratory failure or who are hypoxic prior to intubation are unlikely to benefit. White et al., 2017 provided strong evidence for the benefit of apneic oxygenation in terms of improved SpO_2_ in surgical patients, obese patients, and those undergoing emergency intubation without respiratory failure.^26^ No significant benefit was found in patients with respiratory failure.^26^ Binks et al., 2017 found significant reduction in the incidence of desaturation and critical desaturation when apneic oxygenation was administered.^24^ There was also significant improvement in first-pass intubation success rate.^24^ Similarly, Pavlov et al., 2017 found that apneic oxygenation reduced the relative risk of hypoxemia, along with a significant trend toward lower mortality.^25^

From previous reviews of this intervention, we agree with their findings that there is strong evidence for the use of apneic oxygenation during intubation (excluding certain patient populations).^24^, ^25^, ^26^ There have been relatively few studies of apneic oxygenation in the emergency department (ED); thus, more investigation is warranted, particularly between apneic oxygenation prior to intubation in respiratory and non-respiratory causes.

## LIMITATIONS

This was a review of the literature. All studies were not designed the same way nor did they control for the same outcome measures. There are additional limitations in this literature review. The major limitation relates to the different approaches used to provide apneic oxygenation in terms of preoxygenation and other pre-intubation techniques. Other limitations include the relatively small number of patients, the lack of large clinical trials, the variety of patient clinical conditions and/or comorbidities, and the varied, operationally-defined values of oxygenation desaturation.

## CONCLUSION

Nasal cannula oxygenation during intubation procedures appears to prevent or delay desaturation in all patients except those with primary respiratory failure. Incorporating the use of nasal cannula during intubation has the potential of being integrated into a new standard of care for intubation, whether in EDs or operating rooms. Further research is needed to determine the outcomes and long-term effects of this routine practice, even though the benefits of avoiding hypoxic events during endotracheal intubation are unassailable.

## Figures and Tables

**Table:** Studies included in this review that provide evidence for (*) or against (#) the value of apneic oxygenation with nasal cannula to prevent desaturation during intubation. The characteristics of each study are detailed using a PICO (Populations/people/patient/problem Interventions Comparison Outcome) format.

Study	Patients	Intervention	Comparator	Outcome
Binks et al., 2017*	Systematic review and meta analysis of six studies with 1,822 patients requiring intubation	Nasal cannula during intubation	Without nasal cannula during intubation	All but one study showed a significant risk reduction of oxygen desaturation (RR= 0.76, 95%, CI [0.60 to 0.90], p= 0.002) with significant heterogeneity (I2= 80%, p= 0.0005)
Caputo et al., 2017^#^	Randomized controlled trial in 200 ED patients requiring intubation. Patients were allocated to receive apneic oxygenation (n=100) or standard of care (n=100) by pre-determined randomization in a 1:1 ratio.	Nasal cannula during intubation	Standard of care-No supplemental oxygen during Laryngoscopy	There was no difference in lowest mean oxygen saturation between the two groups (92, 95% CI [91 to 93] in AO vs. 93, 95% CI 92 to 94 in standard of care, p=0.11)
Pavlov et al., 2017*	Systematic review and meta analysis of eight studies with 1,953 patients requiring intubation	Nasal cannula during intubation	Without nasal cannula during intubation	Apneic oxygenation reduced the relative risk of hypoxemia by 30% (95% CI [0.59 to 0.82]). There was a trend toward lower mortality in the apneic oxygenation group (RR of death 0.77; 95% CI [0.59 to 1.02])
White et al., 2017*	Systematic review and meta analysis of eleven studies with 2,078 patients requiring intubation	Nasal cannula during intubation	Without nasal cannula during intubation	Apneic oxygenation during intubation is associated with a reduced risk of desaturation (RR 0.65, p =0.005)
Jaber et al., 2016*	Randomized, controlled, single-center trial with assessor-blinded outcome assessment in 49 patients admitted to the ICU	HFNC [flow = 60 L/min, fraction of inspired oxygen (FiO2) = 100 %] combined with NIV (pressure support = 10 cmH2O, positive end-expiratory pres-sure = 5 cm H_2_O, FiO2 = 100 %)	NIV (PS of 10 cmH2O, PEEP of 5 cm H2O, FiO2 = 100 %)	SpO2 values were significantly higher in the intervention group than in the reference group [100 (95–100) % vs. 96 (92–99) %, p = 0.029]
Riyapan and Lubin, 2016^#^	Retrospective, case controlled study of 29 pre-hospital patients requiring intubation	Nasal cannula during intubation	Without nasal cannula during intubation	Incidence of SpO2 < 90% during intubation 17.2% vs 21.9% in the control group (p = 0.78)
Sakles et al., 2016a*	Observational study of apneic oxygenation on first-pass success without hypoxemia in 635 patients undergoing RSI in the ED	Nasal cannula during intubation	Without nasal cannula during intubation	In the AO cohort the FPS-H was 312/380 (82.1%)
Sakles et al., 2016b*	Prospective comparative study of 127 patients with intracranial hemorrhage requiring intubation	Nasal cannula during intubation	Without nasal cannula during intubation	and in the no AO cohort the FPS-H was 176/255 (69.0%)AO was associated with a reduced odds of desaturation (aOR 0.13; 95 % CI [0.03 to 0.53])
Semler et al. 2016^#^	RCT of 150 ICU patients re-quiring intubation	Nasal cannula during intubation	Without nasal cannula during intubation	Intervention group had an SpO_2_ level of 99% [IQR=96–100%] before intubation and a low-est SpO_2_ of 92% during intubation. 60.5% of patients fell <90% SpO_2_ during intubation. Results were NOT statistically significant
Dyett et al., 2015*	Prospective observational study of 129 patients in the emergency department, ICU and on the wards as part of medical emergency response teams care	Nasal cannula during intubation	Without nasal cannula during intubation	Intervention group without respiratory failure had a significant reduction in incidence of hy-poxemia during intubation (0 of 31)
Miguel-Montanes et al., 2015*	Prospective quasi-experimental study of 101 patients in ICU requiring intubation	Nasal cannula during intubation	Bag valve mask intermittently during intubation	Intervention group maintained a median SpO_2_ level of 100% (range 95–100%) before and during intubation
Vourc’h et al. 2015^#^	RCT of 124 patients with Respiratory Failure requiring intubation	Nasal cannula during intubation	High Fraction-Inspired Oxygen Facial Mask during intubation	Intervention group had a mean SpO_2_ level of 97.1% before intubation and a median SpO_2_ level of 91.5% during intu-bation [IQR=80–96%]. Results were NOT statisti-cally significant
Wimalasena et al., 2015*	Retrospective study of 728 patients requiring intubation by EMS	Nasal cannula during intubation	Without nasal cannula during intubation	Intervention group had a decrease in desaturation rates from 22.6% to 16.5%
Ramachandran et al., 2010*	Prospective RCT of 30 obese patients undergoing surgery	Nasal cannula during intubation	Without nasal cannula during intubation	Intervention group fell below 95% SpO_2_ level at 5.29 min vs 3.49 min in the control
Baraka et al., 2007*	RCT of 34 morbidly obese patients undergoing gastric band or bypass surgery	Nasopharyngeal insufflation during intubation	Without nasopharyngeal insufflation	94% of intervention group maintained an SpO_2_ level of 100% before and after intubation
Taha et al., 2006*	RCT of 30 patients undergoing surgery	Nasal cannula during intubation	Without nasal cannula during	Intervention group maintained an SpO_2_ level of 100% before and during intubation vs comparator who fell below 95% after 3.65 mins
Lee 1998*	RCT of 46 patients undergoing trypanomastoidectomy	Nasal cannula during intubation	Without nasal cannula during intubation	Intervention group had a statistically significant decrease in PaCO_2_ vs comparator at 3 mins
Teller et al., 1988*	Double-blinded, cross-over, RCT of 12 patients undergoing surgery	“Catheter” during intubation	Without “catheter” during intubation	Intervention group maintained an SpO_2_ level of 97% before and during intubation

*AO*, apneic oxygenation; *aOR*, adjusted odds ratio; *CI*, confidence interval; *ED*, emergency department; *EMS*, emergency medical service; *FiO_2_*, fraction of inspired oxygen; *FPS-H*, first-pass success without hypoxemia; *HFNC*, high-flow nasal cannula; *I^2^*, heterogeneity in meta analysis; *ICU*, intensive care unit; *IQR*, interquartile range; *NIV*, non-invasive ventilation; *p*, p-value; *RCT*, randomized control trial; *RR*, relative risk; *SpO_2_*, oxygen saturation.
